# *In Situ* Surface Tailoring with Zwitterionic Carboxybetaine Moieties on Self-Assembled Thin Film for Antifouling Biointerfaces

**DOI:** 10.3390/ma7010130

**Published:** 2013-12-27

**Authors:** Chun-Jen Huang, Ying-Chih Chang

**Affiliations:** 1 Graduate Institute of Biomedical Engineering, National Central University, Jhong-Li, Taoyuan 320, Taiwan; 2 Department of Chemical and Material Engineering, National Central University, Jhong-Li, Taoyuan 320, Taiwan; 3 Genomics Research Center, Academia Sinica, Taipei 115, Taiwan

**Keywords:** surface modification, zwitterionic materials, antifouling properties, self-assembled thin film, biocompatibility

## Abstract

A novel biointerface bearing zwitterionic carboxybetaine moieties was developed for effective resistance to nonspecific adsorption of proteins and blood cells. Self-assembled thin films (SAFs) of (*N*,*N*-dimethylaminopropyl) trimethoxysilane were formed as mattress layers by either vapor or solution deposition. Subsequently, the tertiary amine head groups on SAFs were reacted with β-propiolactone to give zwitterionic carboxybetaine moieties via *in situ* synthesis. The optimal reaction time of 8 h for both preparation methods was verified by static contact angle measurements. According to the X-ray photoelectron spectroscopy, 67.3% of amine groups on SAFs prepared from the vapor deposition was converted to the zwitterionic structures after reaction of β-propiolactone. The antifouling properties of the zwitterionic biointerfaces were quantitatively evaluated in the presence of protein solutions using a quartz crystal microbalance with dissipation, showing a great improvement by factors of 6.5 and 20.2 from tertiary amine SAFs and bare SiO_2_ surfaces, respectively. More importantly, the zwitterionic SAFs were brought to contact with undiluted human blood in chaotic-mixer microfluidic systems; the results present their capability to effectively repel blood cell adhesion. Accordingly, in this work, development of carboxybetaine SAFs offers a facile yet effective strategy to fabricate biocompatible biointerfaces for a variety of potential applications in surface coatings for medical devices.

## 1. Introduction

Biocompatible surface coatings are highly desirable for biomedical devices and implants for *in vitro* and *in vivo* exploitation [1–3]. Biocompatible surface chemistry should allow the devices to perform with an appropriate host response in a specific situation [4]. Unfortunately, nonspecific adsorption on devices is routinely observed and has caused seriously pathogenic problems, such as thrombosis and bacterial infection. Taking an example of thrombosis, its formation is typically initiated when proteins adsorb, subsequently denaturing on surfaces to trigger activation of platelets, and in turn allowing clotting to occur. In this sense, one can rationally envisage that blocking the protein adsorption on the surfaces enables the resisting of the formation of thrombosis and enhancing the biocompatibility of devices [5]. Therefore, surface engineering to modulate nonspecific adsorption is of an increasing interest in the field of biomaterials. Up until now, we have witnessed a great deal of achievements made with the aim of suppressing biofouling [6–9].

Of antifouling materials, hydrophilic poly(ethylene glycol) (PEG) is the most prevalent material regularly used as a surface coating material to resist nonspecific adsorption. Its effectiveness strongly relies on steric repulsion, associated with the energetically unfavorable compression of highly packed PEG while biomolecules approach [10]. Recently, a new class of materials named zwitterionic polymers has attracted considerable attention due to their excellent antifouling properties and stability [6,7,11]. These materials bear both positive and negative ions, resulting in overall interfacial charge balance and strong association with water molecules via ionic solvation. The zwitterionic groups developed in materials include phosphorylcholine (PC), sulfobetaine (SB), carboxybetaine (CB). Nowadays, several efforts moving toward translational medicine have been made with zwitterionic materials. Smith *et al*. [12] modified a catheter with polymeric sulfobetaine (polySB) and showed that the accumulation of thrombotic material on the engineered surface was reduced by >99% even after catheters were exposed to serum *in vitro* for 60 days. Additionally, *in vivo* in a highly thrombogenic canine animal model, device- and vessel-associated thrombus was reduced by 99% [12]. The other remarkable achievement was demonstrated by Jiang’s group [13], showing that an carboxybetaine polymeric hydrogel can resist the formation of a capsule for at least three months after subcutaneous implantation in mice while promoting angiogenesis in surrounding tissue. These works have highlighted the great potential of zwitterionic materials as surface coatings for potent fouling resistance under complex conditions.

In addition to polymer-based materials, self-assembled monolayers (SAMs) provide facile, stable and versatile features, and have been intensively utilized as surface coatings in the fields of biosensing, microelectronics, tissue engineering, drug/gene delivery, and biological signaling and cellular recognition studies [14,15]. Zwitterionic SAMs were firstly introduced by Whitesides’ group [16], unveiling their comparable resistance to protein adsorption with PEG-based SAMs. Furthermore, studies showed that antifouling properties of zwitterionic SAMs are less affected by the changes of temperature, pH and ionic strength [16–20]. More encouragingly, substrates modified with the zwitterionic ligands based on PC [6,17,21] and SB [17,19,22–24] were brought in contact with blood samples, indicating significant reduction in platelet adhesion and prolongation in clotting time. Besides antifouling properties, Jiang’s group [7,25–27] has demonstrated that negatively charged carboxylate groups in CB polymers enables a conversion to functionalizable carboxylic acid groups for immobilization of specific biomolecules. However, to the best of our knowledge, the SAM conjugated with CB moieties has not yet been developed. To follow the trend of zwitterionic materials, in this work, we developed a novel and facile approach to fabricate CB biointerfaces to accomplish the antifouling properties for enhanced biocompatibility.

**Scheme 1.**
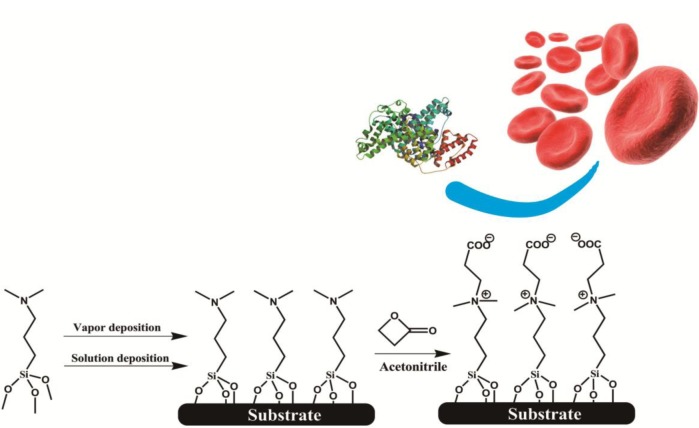
The synthesis route of carboxybetaine self-assembled thin films (CB SAFs) and its antifouling properties.

We employed a two-step synthesis strategy to firstly form a self-assembled silanized thin film (SAF) with tertiary amine (3°-N) head groups, and then a β-propiolactone solution was introduced to react *in situ* with amine groups to give carboxybetaine moieties (Scheme 1). The surface chemistry was characterized by a contact angle goniometer and X-ray photoelectron spectroscopy (XPS) to optimize the reaction conditions and to identify the conversion efficiency of zwitterionic groups. The fouling properties of SAFs were examined by exposing them to protein solutions and undiluted human blood samples. The protein adsorption levels were quantitatively analyzed using a quartz crystal microbalance with dissipation (QCM-D) sensor. To evaluate hemocompatibility of SAFs, a chaotic-mixer microfluidic system was applied to increase the contact probability of blood cells to the surfaces of interest. The adherent blood cells stained with a fluorescent probe were counted under fluorescence microscope. The aim of our study is to develop a strategy for surface modification that can apply onto various hydroxylated surfaces, and to provide a facile yet effective route to establish biocompatible interfaces for a wide spectrum of applications in medical devices.

## 2. Results and Discussion

### 2.1. Characterization of CB SAFs

**Figure 1.**
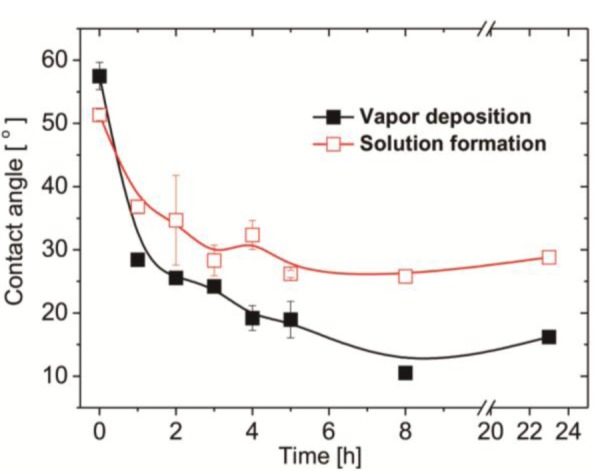
Contact angle measurements for the self-assembled silanized thin film with tertiary amine (3°-N SAF) reacted with β-propiolactone. The 3°-N SAFs were prepared via vapor and solution deposition as described in the experimental section. The *in situ* synthesis of β-propiolactone with tertiary amine head groups on SAFs was carried out in acetonitrile at 4 °C. The contact angles of resultant surfaces were measured at different time points.

**Figure 2.**
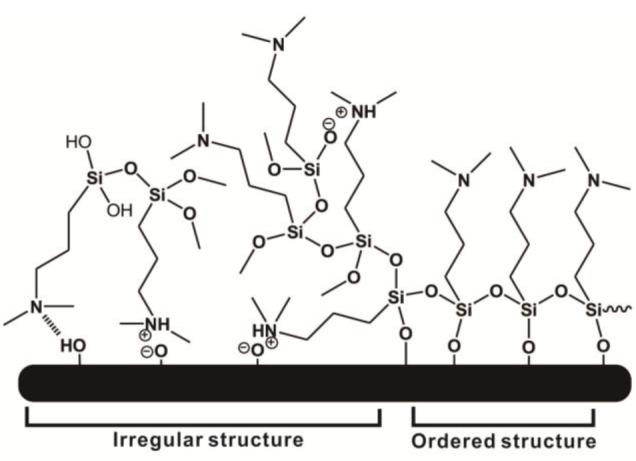
A tertiary amine-terminated silanized thin film on substrate. The films were prepared via either vapor or solution deposition. The complex structure of silane molecules in the film may be caused by hydrogen bonding, electrostatic attraction, and covalent bonding with neighbor silanes, making it irregular and heterogeneous. In contrast, the film may be formed with an ordered and homogeneous structure under a properly controlled condition.

In this study, we employed a two-step approach to fabricate CB-terminated SAFs. Initially, the 3°-N amine silanized films were formed on substrates via either vapor or solution deposition. Afterwards, β-propiolactone solution was introduced for *in situ* synthesis with tertiary amine head groups to give carboxybetaine (CB) moieties on SAFs (Scheme 1). It is well documented that the packing density and orientation of assembling molecules on surfaces are susceptible by the formation approaches, which may change the efficiency of the post-modification with β-propiolactone [14]. Therefore, we monitored the contact angles of substrates after reactions of β-propiolactone at different time points (Figure 1). Before the reaction, the contact angles of the 3°-N amine SAFs prepared via vapor and solution deposition were 57.5° ± 2.2° and 51.4° ± 0.9° (*p* < 0.05, *n* = 3), respectively. The ellipsometric thicknesses of films from vapor and solution deposition were 2.6 ± 0.2 nm and 4.1 ± 0.8 nm, respectively. The contact angles decreased with the reaction time of β-propiolactone to reach minimum values of 10.5° ± 0.2° and 25.8° ± 0.4° at 8 h for SAFs prepared via vapor and solution deposition, respectively. Similar to previous works [6,16,17,22], the decreases in contract angles are attributed to the conversion of 3°-N amine groups to polar zwitterionic CB moieties. Therefore, it is reasonable to propose that the efficiency of the reaction with β-propiolactone on the SAFs prepared via the vapor deposition is significantly higher than that via the solution deposition. This finding may imply the better control in the formation of vapor deposited silanized SAFs, in agreement with the literature [28,29]. The vapor-phase approach was thought to minimize the effects of humidity and silane purity on the reproducibility and quality of silane layer. The polar solvents, such as ethanol used in this study, typically contain a trace amount of water that catalyzes the hydrolysis of siloxane bond formations with substrates and neighboring silanes, leading to multilayer formation and structural irregularities, as illustrated in Figure 2 [14,28,29]. In contrast, the vapor-phase approach operating at moderate temperatures under a vacuum condition enables the anchoring of oligomeric silanes onto substrates directly to form a structurally ordered monolayer and to circumvent the shortcomings of silanization in solution. Therefore, a higher density of CB moieties manifesting as lower contact angles on vapor-deposited silane SAFs, as displayed in Figure 1, is suggested. It is interesting that the contact angles for both cases increased after 24 h incubation compared to 8 h. We suspect that the weakly attached silane molecules, resulting from hydrogen bonding and electrostatic interactions with silicon oxide surfaces [28], were released after long incubation in reaction solvent, *i.e.*, acetonitrile.

**Figure 3.**
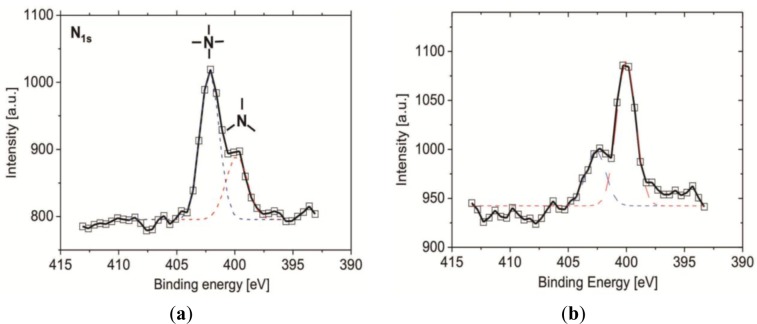
The N 1s spectra of the CB SAF. β-propiolactone reacted with tertiary amine (3°-N) groups on vapor- (**a**) and solution- (**b**) deposited silanized SAFs for 8 h to give the CB moieties. For uncharged 3°-N groups (-N(CH_3_)_2_), the appearance of the N 1s core-level signal is at 399.1 eV; for 4°-N groups, the appearance of the N1s peak component is at 402.3 eV.

In order to further analyze the efficiency of the β-propiolactone reaction, XPS measurement was performed to determine the ratio of quaternary (4°-N) and tertiary amine (3°-N) groups on the substrate after an 8 h reaction with β-propiolactone on vapor- and solution-deposited silane SAFs. Since ionized 3°-N head groups (-N^+^H (CH_3_)_2_) can induce the shift of the binding energy (BE) to BE = 401.3 eV, the substrate was treated with ammonium hydroxide (concentration of 0.1 N, pH = 11) prior to the XPS measurement to deprotonate the 3°-N groups for better deconvolution of the measured spectrum. Ammonium hydroxide has a very low boiling point (*T*_b_ = 37.7 °C at a concentration of 25%) and canthus can be removed easily from the substrates in ultra-high vacuum (below 10–8 Pa) in order for the ammonium hydroxide to no longer remain on the substrates and affecting the XPS measurements. In Figure 3, the presence of 3°-N groups (–N(CH_3_)_2_) can be deduced from the appearance of the N 1s core-level signal at 399.1 eV. The presence of 4°-N groups, on the other hand, is suggested by the appearance of the N 1s peak component at 402.3 eV [30]. From the estimation for the atomic concentrations, 67.3% of the amine groups on the SAF from the vapor-deposition were converted to 4°-N groups, representing the formation of CB moieties. For the SAF from the solution-deposition, the conversion was 25.3%, reflecting the high amount of unreacted 3°-N groups. The result demonstrates that a considerable amount of 3°-N groups remained, likely contributable to the reduced access of β-propiolactone by the steric hindrance of neighboring CB groups and the multi-layer structure of 3°-N SAFs.

### 2.2. Fouling Tests for CB SAF

**Figure 4.**
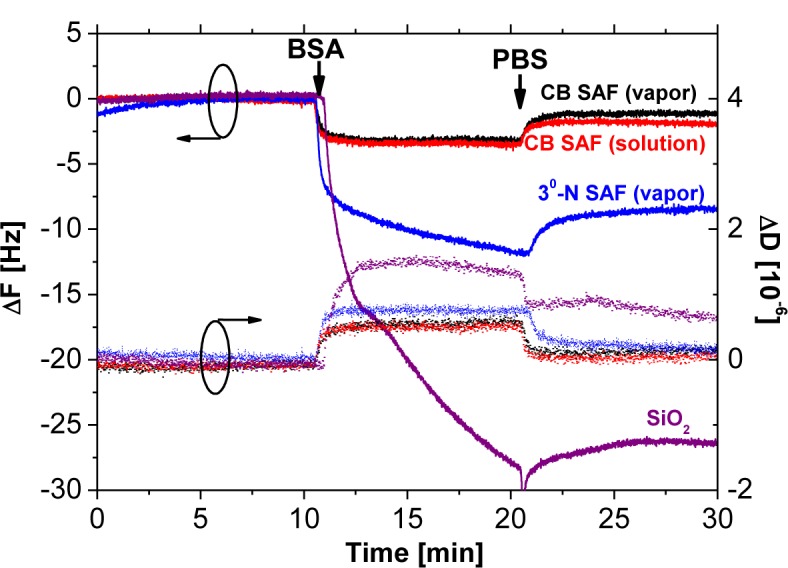
QCM-D measurements of the protein fouling on samples of CB SAF (vapor), CB SAF (solution), 3°-N SAF (vapor) and bare SiO_2_. The bovine serum albumin (BSA) solutions at a concentration of 1 mg·mL^−1^ were brought to contact with surfaces for 10 min, followed by rinsing with PBS.

The QCM-D chips covered with SiO_2_ layers were used for modification of SAFs and monitoring their nonspecific interactions with proteins. Samples of bare SiO_2_, and 3°-N SAFs from vapor deposition were prepared as negative controls for the fouling tests in the presence of BSA solutions at a concentration of 1 mg·mL^−1^ (Figure 4). The CB SAFs were fabricated according to the aforementioned optimal processes. The silane SAFs were prepared from either vapor or solution deposition for the subsequent reaction with β-propiolactone for 8 h, as indicated by CB SAF (vapor) and CB SAF (solution) in Figure 4. The calculation for the adsorbed mass on all surfaces follows the Sauerbrey relation since the changes in dissipation were not significant enough (Δ*D* < 1 × 10^−6^) to take the viscoelasticity of protein adlayers into account [31]. Herein, the changes in ΔF for BSA adsorption on CB SAF (vapor), CB SAF (solution), 3°-N SAF (vapor) and SiO_2_ were 1.3, 1.7, 8.4 and 26.4 Hz, corresponding to the surface densities of Δ*m* = 23.0, 30.1, 148.7 and 467.3 ng cm^−2^, respectively. Clearly, the CB conjugates improved the antifouling properties of substrates from 3°-N SAF and unmodified glass by factors of 6.5 and 20.2, respectively. Owing to the charge balance and strong hydration of CB moieties, the behavior of protein adsorption on CB SAFs is energetically unfavorable, which is inconsistent with previous findings using surface-grafted CB polymer brushes [7,9,13,26,27].

It is interesting that the difference of fouling levels between samples of CB SAF (vapor) and CB SAF (solution) was marginal. We suspect the similar CB densities at the top-most surfaces for CB SAF (vapor) and CB SAF (solution) to render effective repulsion of proteins. The penetration depth of XPS measurements is typically around 10 nm. Thus, the atomic ratio from XPS includes the 3°-N groups underneath the CB layers and cannot reflect the interfacial density of CB moieties. In addition, the contact angle measurement can be affected by surface topography [32]. The higher contact angles measured on 3°-N SAFs from the solution deposition may be considered as the higher surface roughness.

### 2.3. Blood Cell Adhesion in Microfluidic System

**Figure 5.**
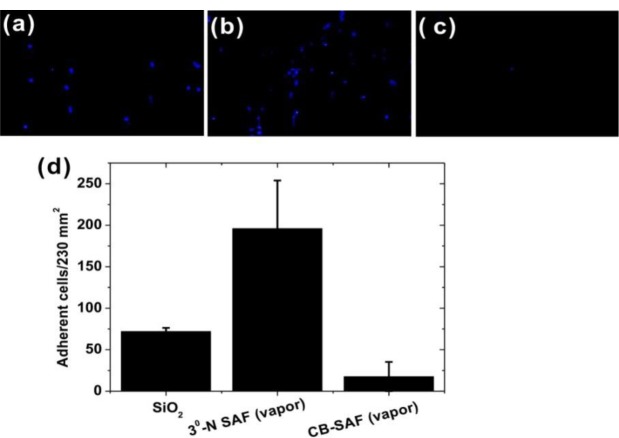
Fluorescence images of adherent blood cells on (**a**) glass; (**b**) 3°-N SAF (vapor); and (**c**) CB SAF (vapor) substrates. The blood samples were flowed in the microfluidic system with chaotic mixer to increase the contact probability of cells to surfaces. After buffer washing, the adherent cells were stained with fluorescent probes and determined using a fluorescence microscope. The quantitative estimation of adherent cell numbers on an image area of 230 mm^2^ is shown (**d**).

The antifouling properties of CB SAFs were challenged with undiluted human blood in a microfluidic system. Since the size of blood cells is in a range of micrometers—for example, erythrocyte with a diameter of 6.5 μm to ~8 μm—the diffusion rate of cells in a laminar flow (Reynolds number, Re < 2000) is too slow to contact the channel walls. Therefore, in this study, we applied a chaotic mixer by creating alternative grooves on the floor of the channel to introduce a transverse component to the flow [33]. By this means, the probability of cell contacts with SAF-tailored surfaces is significantly increased. As seen in Figure 5a–c, the numbers of the adherent cells on bare SiO_2_, 3°-N SAF (vapor) and CB SAF (vapor) were visually quantified under a fluorescence microscope after 2 h challenging with bloods. The quantitative estimation of cell numbers is shown in Figure 5d. Obviously, the blood cells are favorable to adhere on the positively charged 3°-N SAF to a density of 196 ± 58 cells per 230 mm^2^. The adhesion of cells is most likely driven by the interaction between cells and the positively charged 3°-N SAF (pKa = 10.6), as well as pre-adsorbed proteins. The CB SAFs again can resist more effectively than other samples to reduce cell numbers by factors of 4 and 11 from the bare SiO_2_ and 3°-N SAF, respectively.

The capabilities of thiolated and silanized self-assembling surface ligands conjugated with zwitterionic phosphorylcholine (PC) and sulfonbetaine (SB) head groups have been demonstrated to improve hemocompatibility of modified substrates [17,21,22,34]. The most remarkable result discovered in the literature was the great reduction in the adherent platelets on substrates from blood samples. In this work, for the first time, we realized the capability of CB SAFs to resist the adhesion of proteins and blood cells, in turn improving the biocompatibility of substrates. However, the demanding requirement for the “nonfouling” properties of surface coatings was determined by reaching a fouling level of <5 ng cm^−2^ for adsorption of fibrinogen [35]. Below this level, the fibrinogen-mediated platelet adhesion will be eliminated to keep surfaces free of thrombosis. Still, there remains room to improve the antifouling properties of CB SAFs, such as by enhancing the reaction efficiency of *in situ* synthesis with β-propiolactone and considering solvent and concentration effects on SAF formation [21,22].

## 3. Experimental Section

### 3.1. Materials

(*N*,*N*-Dimethylaminopropyl)trimethoxysilane (3°-N silane), sodium dodecyl sulfate, anhydrous ether, bovine serum albumin (BSA), PBS buffer and anhydrous ethanol were purchased from Sigma–Aldrich (St. Louis, MO, USA). Acetonitrile and acetone were acquired from Acros Organics (Bridgewater, NJ, USA). β-propiolactone was obtained from TCI (purity of 98%, Portland, OR, USA). Deionized water was produced from a Millipore water purification system (Billerica, MA, USA). All solid substrates, silicon wafers [Siltec Silicon, single side polished, 500 μm, orientation (100)] and glass slides from a local supplier, were cleaned sequentially by sonication in 0.1% SDS, deionized water and absolute ethanol for 10 min each, followed by drying in a stream of nitrogen. Afterwards, the substrates were transferred to a plasma cleaner (Harrick Plasma, Ithaca, NY, USA) to expose O_2_ plasma twice with a power of 10.5 W for 10 min in order to remove the trace amount of organic components from surfaces. The cleaning process was performed prior to the modification.

### 3.2. Preparation of CB SAFs

For the preparation of 3°-N silanized SAFs on substrates, we adopted two different approaches. One was to use a vapor deposition method where 150 μL of 3°-N silane solution in a small vial was placed in a desiccator along with cleaned substrates. The desiccator was evacuated to reach a pressure of ~10^−3^ torr at room temperature. for 30 min and, then, the system was closed to keep in vacuum overnight. Afterwards, the substrates were removed and washed with copious acetone and dried in a stream of nitrogen. The substrates were cured in an oven at 120 °C for 1 h to complete covalent bonding between silane molecules and substrates. The other method to prepare 3°-N silanized SAFs on substrates was to use solution deposition. The cleaned substrates were immersed in freshly prepared 20 mM 3°-N silane solution in anhydrous ethanol containing extra 2% of water. After 4 h incubation, the substrates were removed and flushed with ethanol and dried in a stream of nitrogen. Similar to the previous method, the substrates were cured at 120 °C for 1 h. The modified substrates were stored in a dry box before use.

For zwitterionization, 10 mM β-propiolactone in acetonitrile was prepared and incubated with 3°-N silane modified substrates at 4 °C within a nitrogen atmosphere for different periods of time. Afterwards, the substrates were flushed with acetonitrile three times and tried in a stream of nitrogen. The resultant substrates were kept in a dry box to avoid contamination from air.

### 3.3. Contact Angle Measurements

A contact angle goniometer (FDS-OCA15 plus, Dataphysics, Germany) was used to measure water static contact angles at solid–liquid interfaces. The droplets were 3 μL from a micro syringe, and the measurements were performed at least three times at random locations on each sample.

### 3.4. Ellipsometric Measurements

Ellipsometric measurements for the films on silicon wafers were performed in ambient with a Gaertner LSE Stokes ellipsometer with a He–Ne laser (λ = 632.8 nm) with a fixed incident angle of 70°. The bare substrates were measured to find the Ns (3.85), Ks (−0.02), and refractive index (*n* = 1.00) of the ambient. The refractive index of the SAFs on the substrates was fixed to *n* = 1.46 and the thicknesses were automatically calculated by the measurement program. All the measurements of the thickness of the samples were performed at least three times at random locations on each sample.

### 3.5. XPS for Element Analysis

The chemical element spectra were detected by a PHI 5000 VersaProbe system (ULVAC-PHI, Chigasaki, Japan) with a microfocused, monochromatic Al KR X-ray (25 W, 100 μm). The takeoff angle (with respect to the surface) of the photoelectron was set at 45°. The pressure of the system is below 10^−8^ Pa using oil-less ultrahigh vacuum pumping systems. A dual beam charge neutralizer (Ar^+^ gun and flooding electron beam) was employed to compensate the charge-up effect. Spectra were collected with the pass energy set to 58.7 eV, and the binding energy measured was normalized against the Au 4f_7/2_ peak at 84.1 eV. The typical data acquisition time was ~30 min; the spectra were processed using the Multipak software package. It should be noted that the substrates for detecting the chemical states of the amine atoms were rinsed with ammonium hydroxide solution at a concentration of 0.1 N prior to the measurements in order to deprotonate unreacted tertiary amine groups for finding the ratio of the quaternary and tertiary amine groups.

### 3.6. Protein Fouling Tests by QCM-D Sensor

The silicon oxide-covered QCM crystal chips (AT-cut quartz crystals, *f*_0_ = 5 MHz) (Q-Sense AB, Gothenburg, Sweden) were cleaned as the aforementioned protocol for substrates. Before the measurement, the chamber was rinsed with PBS and temperature-stabilized at 25 °C. The flow rate of 1 mL·min^−1^ was used. The 1 mg·mL^−1^ of BSA solution in PBS was introduced to have contact with the sensor chip for 10 min, followed by rinsing with PBS. All measurements were recorded at the third overtone (15 MHz), and the data shown here were normalized to fundamental frequency (5 MHz) by dividing the overtone number. In addition, since BSA is a globular and relatively rigid molecule, the increased mass on the chip is well related to changes in frequency of the oscillating crystal through Sauerbrey relationship:

$$\Delta m_{Sauerbrey}=\frac{C_{QCM}\times \Delta f}{n}$$ (1)

where Δ*m*_Sauerbrey_ represents mass adsorbed on the quartz sensor, Δ*f* is resonance frequency, *C*_QCM_ is the mass-sensitivity constant (=17.7 ng·cm^−2^·Hz^−1^ at *f* = 5 MHz), and *n* is the overtone number (=1, 3, 5 and 7) [36–38].

### 3.7. Human Blood Cell Adhesion Tests in Microfluidic System

For the manufacturing of a chaotic-mixer microfluidic system, the channel with a dimension of L:W:H = 230 mm:41.5 mm:5.5 mm was created by placing a spacer of a double-sided adhesive tape (3M, St. Paul, MN, USA) with a channel shape between a glass cover slide and a poly(methyl methacrylate (PMMA) substrate. The alternative grooves with a dimension of L:W:H = 1.5 mm:150 μm:50 μm on the PMMA substrate were fabricated by a commercial CO_2_ laser scriber (M-300, Universal Laser Systems, Scottsdale, AZ, USA) [39,40]. The groove pattern was designed using CorelDraw (Corel, Ottawa, ON, Canada) and then transferred to the laser scriber for direct machining on the PMMA substrate. The surface modification on glass cover slides was accomplished as described previously. After the assembly of the microfluidic system, the solution was introduced using a syringe pump (Harvard PHD 2000, Instech Laboratories, Plymouth Meeting, PA, USA) at a flow rate of 0.5 mL·h^−1^. For the blood fouling test, the blood samples were flowed over the modified surfaces for 2 h, followed by PBS washing. The resultant samples were stained with fluorescent probes (CellTracker, Invitrogen, Carlsbad, CA, USA) for observation and analysis under a fluorescence microscopy (Leica AF 6000, Wetzlar, Germany).

## 4. Conclusions

In summary, a novel approach to developing a zwitterionic interface based on carboxybetaine (CB) moieties to resist adsorption of proteins and blood cells has been carried out. The substrates were firstly silanized with tertiary amine-terminated self-assembled thin films (3°-N SAFs) via vapor or solution deposition. The synthesis of β-propiolactone molecules was sequentially performed over a period of 23 h, showing that after 8 h reaction, the contact angles of CB SAFs reached the minimum. Moreover, the 3°-N SAF via the vapor deposition offers higher reaction efficiency with β-propiolactone, manifesting as a higher degree of hydration. The X-ray photoelectron spectroscopic measurements presents the conversion rate to the CB moieties as approximately 67.3% and 25.3% on 3°-N SAF via the vapor and solution deposition, respectively. More importantly, the fouling tests with protein and blood samples on CB SAFs revealed the significant improvement in biocompatibility. It is believed that this approach of surface engineering with CB SAFs offers great potential in modification of medical devices for future *in vivo* therapeutic and diagnostic applications.
